# Convolutional Neural Networks for Real Time Classification of Beehive Acoustic Patterns on Constrained Devices

**DOI:** 10.3390/s24196384

**Published:** 2024-10-02

**Authors:** Antonio Robles-Guerrero, Salvador Gómez-Jiménez, Tonatiuh Saucedo-Anaya, Daniela López-Betancur, David Navarro-Solís, Carlos Guerrero-Méndez

**Affiliations:** 1 Unidad Académica de Ingeniería, Universidad Autónoma de Zacatecas, Zacatecas 98000, Mexico; aroblesp@uaz.edu.mx (A.R.-G.); jimenezs@uaz.edu.mx (S.G.-J.); danielalopez106@uaz.edu.mx (D.L.-B.); david.navarro@uaz.edu.mx (D.N.-S.); 2 Unidad Académica de Ciencia y Tecnología de la Luz y la Materia, Universidad Autónoma de Zacatecas, Campus es Parque de Ciencia y Tecnología QUANTUM Cto., Zacatecas 98160, Mexico; tsaucedo@uaz.edu.mx

**Keywords:** beehive acoustic classification, beehive monitoring, precision apiculture, precision beekeeping, convolutional neural networks

## Abstract

Recent research has demonstrated the effectiveness of convolutional neural networks (CNN) in assessing the health status of bee colonies by classifying acoustic patterns. However, developing a monitoring system using CNNs compared to conventional machine learning models can result in higher computation costs, greater energy demand, and longer inference times. This study examines the potential of CNN architectures in developing a monitoring system based on constrained hardware. The experimentation involved testing ten CNN architectures from the PyTorch and Torchvision libraries on single-board computers: an Nvidia Jetson Nano (NJN), a Raspberry Pi 5 (RPi5), and an Orange Pi 5 (OPi5). The CNN architectures were trained using four datasets containing spectrograms of acoustic samples of different durations (30, 10, 5, or 1 s) to analyze their impact on performance. The hyperparameter search was conducted using the Optuna framework, and the CNN models were validated using k-fold cross-validation. The inference time and power consumption were measured to compare the performance of the CNN models and the SBCs. The aim is to provide a basis for developing a monitoring system for precision applications in apiculture based on constrained devices and CNNs.

## 1. Introduction

The current research in precision apiculture indicates an interest in developing automatic devices to monitor and analyze the health of honey bee colonies. Some monitoring systems are specifically designed to analyze acoustic patterns. As several studies indicate, bee acoustics can provide important information about their condition, and the sound of regular activity can be affected by numerous factors, such as the absence of a queen [1,2,3,4,5,6], the activity before the swarming phenomenon [7,8,9,10,11,12,13,14,15], the exposure of bees to chemicals [16,17,18], and even the presence of a human, which can lead to stress in the colony members and result in a hissing sound as a warning. The use of dedicated devices is expected to address some problems presented in traditional beekeeping; this includes reducing invasive inspections that cause stress to the colony members, minimizing the loss of the queen due to poor beekeeping practices, and most importantly, detecting critical events that can cause the death of bee colonies.

Developing a monitoring system based on a CNN for constrained devices presents significant challenges, including energy efficiency, computing power, and limited diversity of samples in datasets. Monitoring honey bee colonies in real-life conditions is challenging because most apiaries are located in remote areas without access to uninterrupted power sources. For monitoring honey bee colonies, various research projects have suggested system configurations using single-board computers (SBCs) such as the Raspberry Pi [4,6,17,19,20,21,22,23,24]. A monitoring system could perform several tasks, including monitoring and analyzing the parameters of bee colonies, storing data, and identifying the state or condition of bee colonies, among others. Additionally, some monitoring systems include additional sensors to measure parameters that can provide information about the beehive state, such as temperature, humidity, and weight; however, the use of additional sensors makes the system more power-demanding. In some investigations, the use of solar cells may not be the optimal solution for powering monitoring systems [20,25]. Hence, power consumption becomes a primary concern.

CNNs are deep learning algorithms that process input images to learn visual features. In precision beekeeping, the most common type of image used to train CNN models is spectrograms of acoustic samples [26]. However, obtaining acoustic patterns and accurately correlating them with the actual colony state is challenging; it is essential to avoid frequent inspection to prevent affecting regular activity in the beehive. The dataset size leads to other problems, as having a significant amount of images is necessary to prevent overfitting of the CNN models [27], provide diversity, and enhance the generalization capacity. Furthermore, CNNs can require more computational resources in the training step compared to conventional machine-learning models. Therefore, the inference time can be longer depending on the complexity of the CNN model. The computational nature of CNN models may limit the development of dedicated devices for monitoring due to hardware constraints.

This study examines various aspects of implementing CNNs for classifying bee acoustic patterns on resource-constrained hardware. For this purpose, ten CNN models were selected based on their reported FLOPS as a measurement of their computational complexity. Most of the selected CNN architectures were developed specifically for use on mobile devices. The CNN models were implemented using PyTorch and Torchvision libraries in Python. Acoustic patterns from five colonies of Carniola honeybees in different conditions were recorded for 30 s. For the training step, the original dataset was segmented into samples of different durations: 10, 5, and 1 s. The aim is to analyze how the duration of the sample impacts the inference performance. The CNN models were trained on a desktop PC and then transferred to the SBCs (the Raspberry Pi 5, a Nvidia Jetson Nano, and Orange Pi 5). The inference time and the power consumption were measured to compare their performance. The goal is to select the CNN model with the best performance and the SBC with the best energy efficiency to construct a monitoring system capable of inferring the state of bee colonies.

The rest of the paper is organized as follows: Section 2 presents an overview of the applications of CNNs on precision apiculture. Section 3 describes the dataset, the characteristics of the SBCs, the selected CNN models, the hyperparameter optimization, and performance evaluation and validation. Then, the results and discussion are presented in Section 4. Finally, Section 5 provides the conclusions and future work.

## 2. CNN in Precision Apiculture

Recent advances indicate that convolutional neural networks (CNNs) have proven to be highly effective in detecting the health of honey bee colonies through the classification of acoustic patterns [14,20,28,29,30,31]. Furthermore, some studies have shown that the prediction performance achieved using CNNs is superior to that of conventional machine learning models [6,28,29]. The following paragraphs review the most significant research applications of CNNs in the field of precision apiculture.

One of the first studies published about CNN models to detect the presence of bees was conducted by Kulyukin et al. [28]. Their study analyzed spectrograms of audio samples and categorized them into bee buzzing, cricket chirping, and ambient noise. Two CNN architectures were developed to classify the spectrograms: SpectConvNet, which was designed to work with spectrograms, and RawConvNet, which was intended for raw audio waveforms. The CNN models were compared against standard machine learning models such as logistic regression, k-nearest neighbors (KNN), support vector machine (SVM), and random forest (RF). The RawConvNet model achieved the best performance, with an accuracy exceeding 95% in two datasets. The same dataset was used in another study developed by Truong et al. [32]; they proposed a new methodology based on a CNN and Mel spectrograms called Mel-CNN-GRU using PyTorch; (v2.0.1) they claim that they achieved superior performance compared with previous CNN models mentioned before.

Another study to identify the sounds of bees from environmental sounds of animals, rain, and others was developed by Nolasco et al. [30]. They compared the performance of a CNN model against an SVM model. They created a dataset with acoustic samples of the projects OSBH and NU-Hive. In this instance, the SVM classifier outperformed the CNN model. Jaehoon et al. [29] performed a study to identify the sound of bees and non-bees; they compared conventional machine learning models such as SVM, RF, and XGBost with CNN architectures, shallow CNN, and VGG-13. The identification performance of bee sound patterns was outstanding, with a precision of 0.99 in all models. However, the models perform poorly in recognizing non-bee sounds.

In order to identify the difference between regular activity and swarm activity, Zgank [14] developed a classification model based on deep neural networks using acoustic samples of the OSBH project dataset. The deep neural network outperformed hidden Markov models, achieving an accuracy value of 0.94. Dimitrios et al. [6] developed another method to detect swarm activity using a CNN architecture called U-Net; they compared its performance against conventional machine learning models such as SVM and KNN. They classified spectrograms of acoustic samples recorded in two scenarios: 5 and 10 days before swarming takes place. The accuracy values showed that KNN performed slightly better than U-Net. Hunter et al. developed a CNN model [31] to predict the swarming state in bee colonies. The audio data were categorized in days before the swarming event. The CNN models were trained with short-time Fourier transform (STFT) spectrograms and Mel spectrograms. According to their findings, the swarming event can be efficiently predicted at an early stage.

To predict the queenless state, Terenzi et al. [33] conducted a study to analyze methods for representing acoustic patterns. These methods included STFT spectrograms, Mel spectrograms, Mel frequency cepstral coefficients (MFCC), Hilbert Huan transform, discrete wavelet transform, and continuous wavelet transform. Each representation was used as input for a CNN; according to their findings, the best performance was achieved using STFT spectrograms.

Researchers have proposed other interesting applications of CNNs, such as classifying the comb cells to estimate the bee colony health, nutrition status, queen quality, and honey yield [34]; analyzing the traffic of bees at the entrance of the hive using images [20]; identifying the presence of varroa mites among the colony members using a Raspberry Pi 4 and a tensor processing unit [21]; predicting the strength of bee colonies and classifiers in low-disease, medium-disease, and high-disease scenarios [35]; optimizing CNN models to detect the presence of queen bees in the Arduino nano using a low-power microcontroller [36]; preprocessing spectrograms to enhance the performance of CNN architectures [37].

## 3. Materials and Methods

### 3.1. Dataset Description

The dataset comprises acoustic samples from five beehives of Apis Mellifera Carnica (italics) recorded from 22 March to 6 May 2018. The beehives were in crop fields in Zacatecas, Mexico, far from the city. Beehives with different characteristics were selected from an apiary comprising 26 beehives by a professional beekeeper. Two of the beehives were healthy and free of mites or diseases, with a productive queen and a large population of around 60,000 bees each; only these beehives had a super with honey stored. Two other beehives were also healthy, free of mites or diseases, and had a productive queen, but these beehives had a lower population compared to the first two, with around 40,000 bees each. A fifth beehive did not have a queen bee and had a reduced population of around 30,000 bees. Electret microphones were placed inside the beehive over the frames in the brood chamber to avoid interfering with the colony members. The acoustic samples were recorded for 30 s at 10 min intervals, resulting in 144 samples per day; a total of 2147 samples were recorded over 15 days. The sampling frequency was set to 4 kHz in compliance with the Nyquist theorem; this frequency is sufficient since most buzzing sounds in a beehive fall within the low-frequency range of 100–600 Hz [38,39,40,41].

### 3.2. Single-Board Computers

The CNN architectures were tested using three devices: a Raspberry Pi 5 (RPi5), an Orange Pi 5 (OPi5), and a Nvidia Jetson Nano (NJN). Table 1 summarizes the most relevant features of the SBCs. The RPi5 is the most popular SBC with excellent operating system (OS) support and a broad community of developers; however, comparing the hardware specifications, the RPi5 is the most limited SBC, featuring a quad-core processor. On the other hand, the OPi5 features an octa-core processor, which can significantly speed up data processing. The NJN incorporates a graphics processing unit (GPU) with CUDA cores; the acronym CUDA stands for Compute Unified Device Architecture. The CUDA cores enable the CNN models to utilize the GPU to accelerate the inference process.

The SBCs were equipped with heatsinks and fans for cooling; in the RPi5 and the NJN, the fan turns on when the temperature exceeds a threshold value. In the OPi5, the fan always remains active, which will impact the power consumption. The SBCs were powered by a USB-C power supply (5 V, 5 A). The NJN can be powered by micro-USB and DC barrel jack; when using micro-USB, it is recommended to use a power supply that can deliver 2–3.5 A, and when using the barrel jack, it is recommended to use a power source that can supply 5 V–4 A for stressful workloads. A USB-C to micro-USB adapter was used with the same power supply to power the NJN.

A USB-C multimeter TC66C was used to quantify the power consumption during the inference process. The principal features of the meter are displayed in Table 2. An external power supply can be used to power the meter, preventing the system from drawing power from the USB port used for measurement. The sampling rate is 1 Hz, which is sufficient to measure power consumption accurately.

### 3.3. Spectrograms of Acoustic Samples

**Figure 1:**
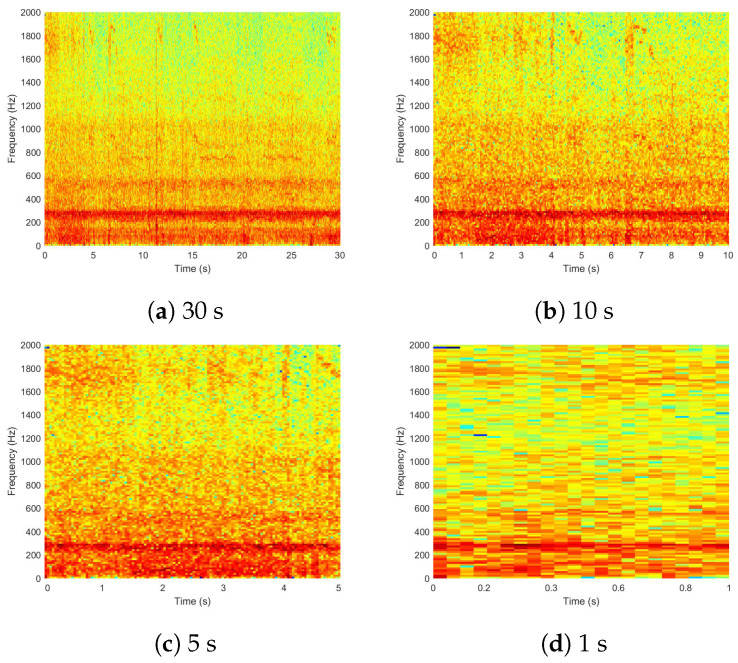
Spectrograms of one acoustic sample segmented in different sizes to determine the effect on the inference performance.

A spectrogram is a visual representation of the frequencies of sound samples as they change over time. In precision apiculture, spectrograms have been widely utilized to identify swarming [7,8,31,42,43], the presence of the queen [5,44,45], and the exposure of bees to air pollutants [17,18], among others. The impact of the spectrogram on inference performance was analyzed by creating datasets of acoustic samples segmented into different durations. The original acoustic samples were recorded at 30 s long, looking for a balance between preprocessing time, storage space, and the information carried out by the sample. The acoustic samples were segmented based on the most commonly used sizes by researchers: 10, 5, and 1 s [6,10,13,26,28,42,46]. Figure 1 shows an example of the spectrograms. In Figure 1a, most of the energy signal is concentrated within the frequency range of 200–400 Hz, which is typically found due to beehive activity, and with less intensity in a narrow band of 500–600 Hz. As the acoustic sample duration decreases, the spectrogram resolution also decreases. In the 1 s spectrogram, the narrow band in the 500–600 Hz range is barely noticeable. Four datasets were created, each consisting of 1000 randomly selected spectrograms. Also, the labels and tick marks in the spectrograms were removed.

### 3.4. CNN Models

**Figure 2:**
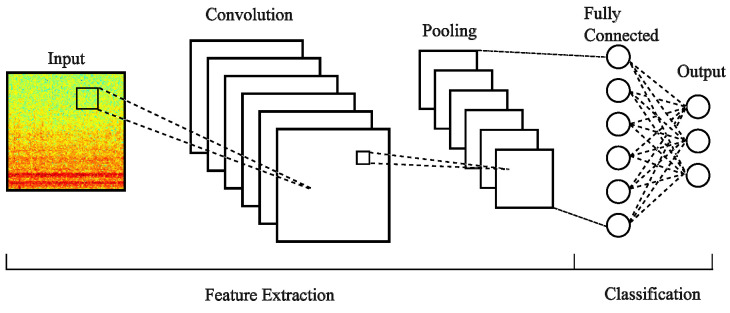
Schematic diagram of a basic CNN architecture.

CNN arquitectures are a class of deep learning networks inspired by how human and animal brains work [47]. CNNs are extensively used in applications such as speech processing, natural language processing, and computer vision, among others [47,48]. Figure 2 illustrates the basic representation of how CNNs work. Normally, CNN architectures comprise five stages: input, convolution, pooling, a fully connected layer, and an output layer. These stages are grouped into two categories: feature extraction and classification. The first stage is the input layer, which involves resizing an image to a specific size and then using it to feed a convolutional layer. Each convolutional layer contains several filters (kernels) that are convolved with the input, producing a two-dimensional representation of the image. The pooling layer involves sliding a two-dimensional filter to reduce the spatial size of the representation while trying to preserve the representative information, and the resulting output is known as a feature map or activation map, marking the completion of the feature extraction process. The next step usually involves a fully connected layer, similar to a regular neural network; the feature map is converted into vector form and passed to the fully connected layer; in the final layer, output neurons corresponding to each category of the dataset are used to generate the classification scores that represent the probability for a given instance.

The CNN models used in this research are part of the ML library PyTorch and TorchVision, which is built in Python. All the architectures in the library are pre-trained. This means that the CNN models were previously trained on a large-scale dataset with a specific objective. The weights are used as a starting point for training a model for a new classification task, which reduces the computational cost of the training process.

Ten CNN models were selected considering the FLOPS (Floating Point Operations Per Second), which, in deep learning, represents a measure of the model complexity and computational cost. Higher FLOPS values usually indicate a more complex model, leading to more extended training and classification times and, therefore, a less energy-efficient model. However, a more complex model can benefit from a high inference performance. Most of the models selected have a low FLOPS value, as they were designed to run on mobile devices. Additionally, the ConvNext model, with a high FLOPS value, was introduced to contrast the performance of the models with low FLOPS values. Table 3 presents the CNN models and version information.

### 3.5. Transfer Learning and Data Argumentation

In transfer learning, the information of the learning process of a source domain is transferred to the target domain to reduce the learning cost and improve the performance of the target learners [49], i.e., it is a process that uses a pre-trained CNN architecture with a different objective to solve a new classification task, and this procedure aims to reduce the training time. This research used the fine-tuning method since the dataset size is relatively small and the images are unrelated to the previous training dataset. The fine-tuning process involves updating all the weights of the neurons in the CNN model during the training process while preserving the architecture of the CNN model. Also, the last layer of the neural network is modified according to the number of classes of the problem—in this case, the identifier of the beehives. Data argumentation is used in deep learning to artificially increase the dataset size and provide diversity when the quantity of samples is limited (e.g., medical images); the aim is to enhance the capacity of the CNN model to generalize. Data argumentation works by applying a random transformation to the existing image; these transformations could include geometric transformations such as axis flipping, argumentation in image color channels, cropping, rotation, and noise injection, among others [50]. The CNN models tested will classify spectrograms with specific features such as sampling frequency and duration of the audio samples; therefore, if a new spectrogram needs to be classified, it will have the exact specifications. At this research stage, the transformations applied to the images were resize, crop, and horizontal flip.

### 3.6. Hyperparameter Optimization

The hyperparameters are parameters that can be adjusted to control the learning process and optimize the performances of CNN models [51]. The Optuna framework in Python was used to search for the best hyperparameters. Optuna is an automated search tool that determines the combination of hyperparameters for a model that optimizes the prediction performances [52]. By default, Optuna frameworks use Bayesian optimization. However, hyperparameter optimization can be performed using other methods, such as random search or grid search. Bayesian optimization builds a probabilistic model of the objective function, called the surrogated function; then, an acquisition function is used to select the next potential hyperparameters based on the current posterior distribution [53,54]. Optuna uses a pruning strategy to reduce the time required for hyperparameter optimization [52]. If, during a single execution of the objective function with a specific set of hyperparameters, the model does not meet a performance minimum after several epochs, the set of hyperparameters is discarded.

### 3.7. Performance Evaluation and Validation

The accuracy metric was used to assess the predictive performances of the CNN models. Accuracy represents the fraction of samples that were correctly classified from the total of samples. In order to validate the results, a k-fold cross-validation methodology was implemented; the technique involves segmenting the dataset into k-folds, where one fold is removed for validation, and the rest of the dataset is used to train the CNN model. The performance of the model is assessed using the accuracy metric; subsequently, the hold-out part of the dataset is reintegrated, and a new fold is used for validation. The procedure is repeated k times, and the performance is calculated as the average of the accuracy values from all the folds.

### 3.8. Software

The SBCs come with an operating system, each supporting specific versions of Python packages and libraries. In the NJN, Ubuntu 20.04 was installed; the OS image can be found on GitHub [55] and includes the PyTorch and TorchVision libraries to implement the CNN models and CUDA libraries to enable GPU usage. It is important to note that this is not the official OS provided by Nvidia; the official software is the JetPack SDK, and the last version that supports the NJN is 4.6.5. However, some conflicts were found when installing the Pytorch library in the JetPack SDK. In the case of RPi5 and OPi5, the OSs are based on versions of the Debian distribution. The CNN models were implemented using the framework PyTorch for deep learning, which is based on the Torch library; the pre-trained CNN architectures and weights are included in the Torchvision package. In every SBC, the libraries were installed through Pip (the package installer for Python). The specific Python, PyTorch, and Torchvision versions used in each SBC are shown in Table 4. The CNN models were previously trained on a desktop PC with an Intel Core i5 13600k, 32 GB of RAM, and an Nvidia RTX 3060 GPU. The PyTorch (v2.0.1) and Torchvision (v0.15.2) libraries were installed on the PC through the Anaconda distribution v23.7.2 and Python v3.11.4.

## 4. Results and Discussion

### 4.1. Hyperparameter Search

For hyperparameter optimization, four datasets (D30, D10, D5, and D1) were created, each containing spectrograms of acoustic samples with durations of 30, 10, 5, and 1 s. Each dataset consists of 1000 randomly selected spectrograms. A total of 40 studies were conducted; that is to say, each CNN model was optimized using four datasets. Hyperparameter optimization is a time-consuming task due to the high computational cost; to accelerate the process, the search space was limited to the learning rate, the batch size, and the optimizer. The learning rate is considered the most important hyperparameter; it determines the step size in each iteration, enabling the objective function to converge [56]. The search for the learning rate was in the range of 0.0001–0.01. The optimizers analyzed were SGD (stochastic gradient descent) with momentum and Adam (adaptive moment estimation). The SGD optimizer is widely used in machine learning [57] and is known for its simplicity and effectiveness; on the other hand, the Adam optimizer is one of the most popular methods for training neural networks [58]. The advantage of Adam against SGD is rapid progress in the training step [59]. Finally, the analyzed batch sizes were 64, 32, and 16. The search space for the Optuna algorithm is outlined in Table 5.

The Optuna package was configured with the following values: 40 trials (a single execution of the objective function) and 20 epochs, reaching an excellent fit with those values. The momentum is a hyperparameter used in the SGD optimizer; this value was set at 0.9; the momentum helps to accelerate SGD in a suitable direction and dampens oscillations [60]. The results of the hyperparameter search are shown in Table 6. For every CNN model, the learning rate varies slightly across the different datasets, while the optimizer is the same in most cases. The batch size is variable in models such as MNASNet, RegNet, and SqueezeNet and could have minimal impact on performance.

### 4.2. K-Fold Cross Validation

**Figure 3:**
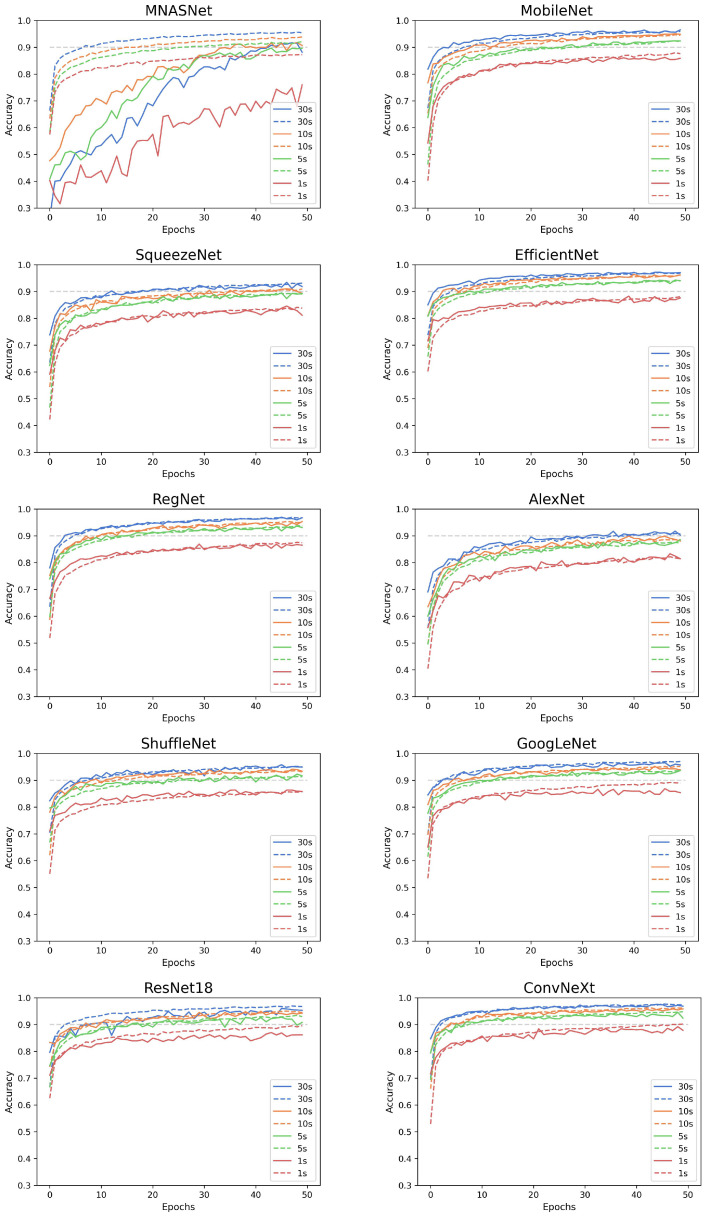
Accuracy curves for the k-fold cross-validation of the CNN models: the dashed line represents the validation step, while the solid line represents the training step.

In the cross-validation, the dataset was divided into five folds, with 80% for training and the remaining 20% for validation. The cross-validation was performed using the four datasets. The CNN models were trained for 50 epochs with the hyperparameters computed previously. Figure 3 shows the average accuracy through the cross-validation process for training (solid lines) and testing (dashed lines); the horizontal gray dashed line marks 0.9 accuracy. The CNN models trained with the D30 dataset exhibit similar behavior, with accuracy increasing rapidly, achieving a value of 0.9 before ten epochs, except for the models SqueezeNet, AlexNet, and MNASNet. As the duration of the spectrogram decreases, the accuracy curves also decrease in most models. The curves of the D10 and D5 datasets remain very close to the D30 dataset, and before reaching 50 epochs, the CNN models overcome an accuracy of 0.9. The accuracy curves for the D1 dataset are all below the 0.9 value, except for the ConvNeXt model, which is the most complex CNN model.

To compare the performance of CNN models, we extracted the maximum accuracy values from the validation step, which are outlined in Table 7. Using the D30 dataset, the EfficientNet and ConvNeXt models achieved the highest accuracy values (0.97), while the lowest values were obtained with MNASNet and AlexNet (0.91). In every step-down through the datasets, the accuracy decreases by a percentage. However, the EfficientNet and ConvNeXt models preserve the best overall performance, achieving an accuracy value above 0.88 with the D1 dataset.

### 4.3. Performance in the SBC

In order to determine the performance of the SBCs, the CNN models were previously trained on a desktop PC using the D30 dataset and 2147 spectrograms per class. The training stage consisted of 50 epochs; the CNN model with the maximum accuracy at a particular epoch was saved in a pth file, which is a type of dictionary only containing the learned parameters from the training process. The pth files were transferred in each SBC and loaded into a new CNN model. In the study, 200 random spectrograms were classified to compare the power consumption and inference time. The NJN has two power modes, 5 and 10 W (MAX); this works by limiting the maximum frequency of the CPU and the GPU [61]. The NJN was powered by the micro-USB port using an adapter. It is important to note that a warning about the power supply was displayed when the NJN was under a high workload; however, the classification task continued without interruption. In the OPi5 and the RPi5, the Wi-Fi was deactivated to reduce the power consumption; the NJN does not include the Wi-Fi module.

#### 4.3.1. Inference Time

**Figure 4:**
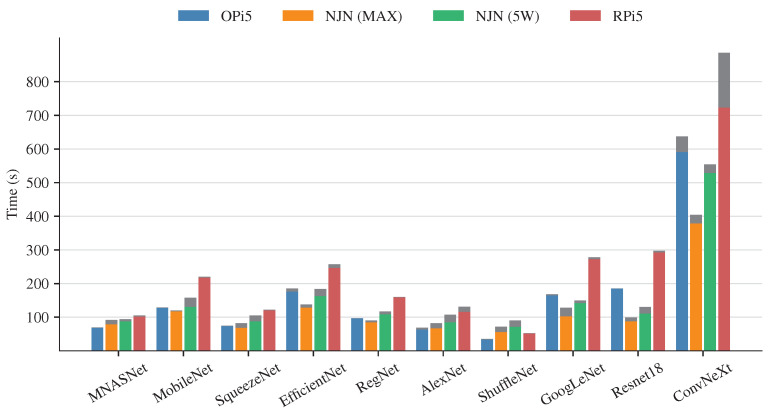
Inference time of the CNN models.

The inference time was measured in two scenarios: the first scenario involved the execution of the entire code, including the loading of the model parameters and the inference step, while the second scenario only considered the inference step. The codes were executed three times, and the average values are shown in Figure 4. The color bars represent the inference time, and the gray bars indicate the time to load the parameters into the new CNN models. Analyzing the inference times, the ConvNeXt model has the longest inference time due to its computational complexity; the intention was to contrast the CNN models with those with less computational complexity. The models with the shortest inference time are ShuffleNet and MNASNet; however, the results are very similar to the rest of the CNN models. When comparing the inference time of the Single-Board Computers (SBCs), the Nano Jetson (NJN) with the MAX power mode achieves faster classification times in most cases. However, for the MNASNet and ShuffleNet models, the OPi5 performs better. This comparison does not consider the time required to load the parameters of the models. Also, the results achieved by the NJN in the 5W power mode are very close to the MAX power mode, except in the ConvNeXt model, which presents a significant difference. The SBC with the slowest inference times is the RPi 5. An exception to highlight is the inference time achieved by the ShuffleNet model, which is lower than the NJN and very close to the time achieved by the OPi 5. Finally, the time to load the parameters of the models is very short in OPi5 and, in some cases, minimum compared with the inference time.

#### 4.3.2. Power Consumption

**Figure 5:**
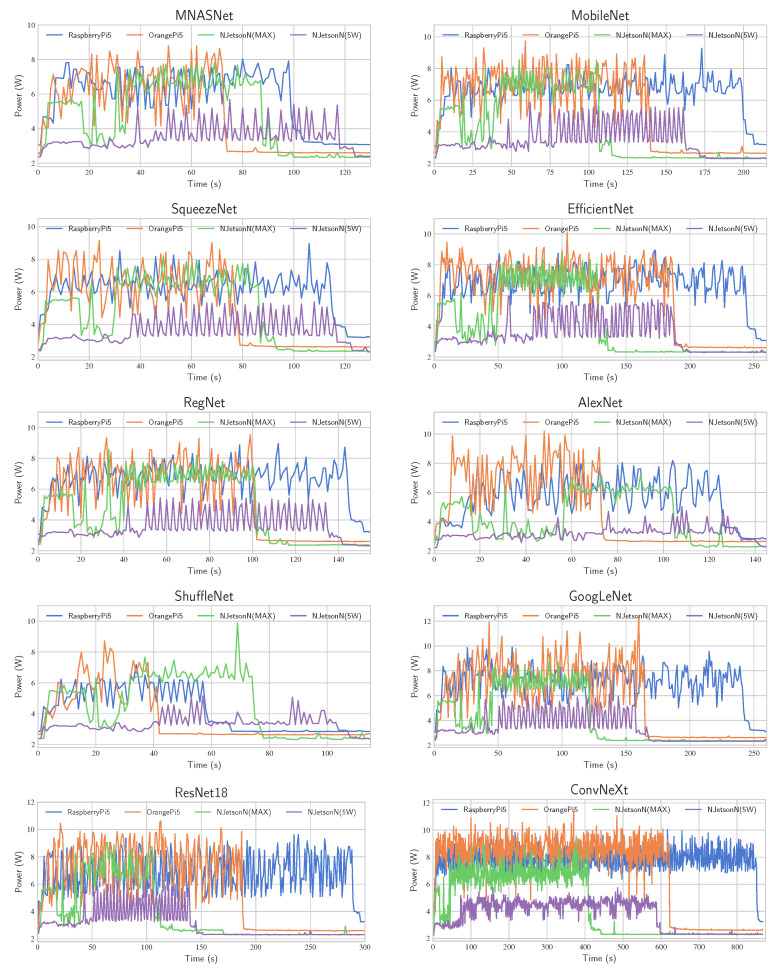
The power demand in the SBCs in the inference step.

The plots in Figure 5 show the power required by the CNN models during the workload, specifically during the execution of the classification task. In most cases, the highest peaks are of the OPi5 computer, followed by the RPi5 and the NJN in MAX power mode. The CNN models stress the SBCs in different modes; for example, in the OPi5, the models AlexNet and ConvNeXt have a superior power demand compared to other SBCs. The maximum required power in the NJN in the 5 W mode is considerably smaller than the other SBCs. However, the same CNN model does not require double the inference time of the NJN in MAX mode. Analyzing the initial stages of the NJN plots in both power modes (MAX and 5 W), it becomes evident that there is a variable workload before the execution before the inference step. During this period, the power demand is low, but the total inference time is increased, especially in the 5 W mode. The idle power can be observed at the end of the plots. The NJN has the lowest idle power demand in both power modes, at approximately 2.4 W. The highest value is presented in the RPi5 at 3 W and the OPi5 at 2.7 W. It is important to note that these values are measured using a display; however, the power consumption can be significantly reduced without a display for the OPi5 to 1.85 W and NJN to 1.4 W; in the case of the RPi5, the power demand is almost the same, i.e., about 2.7 W.

**Figure 6:**
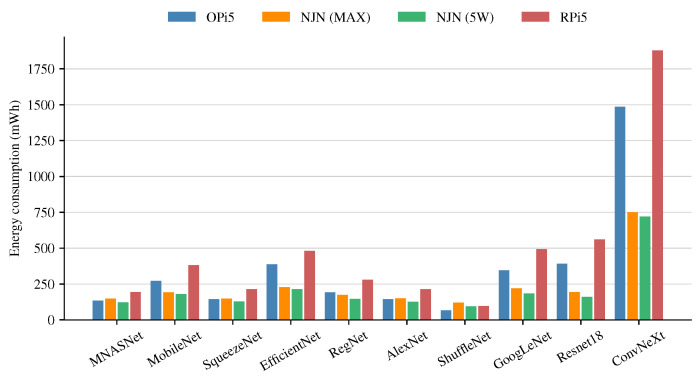
Energy consumption of the CNN models.

Figure 6 presents a comparison of the energy consumption of the CNN models. When comparing the energy consumption of the NJN in both power modes, the energy consumption is almost the same and slightly lower in the 5 W mode. Contrasting the results with the inference time is not observed as a lineal behavior, meaning that doubling the energy consumption does not result in half the inference time. On the other hand, the OPi5 consumes less energy than the RPi5, even though the OPi5 uses an octa-core processor. Also, the energy consumption of the OPi5 is very close to the consumption of the NJN in MAX mode. Finally, the RPi5 has the highest energy consumption, except for the case of the ShuffleNet model. Comparing the CNN models, the ShuffleNet model has the lowest energy consumption; even without using a GPU, the power consumption is very similar to the NJN in both power modes. Finally, the energy consumption of the ConvNeXt model is significantly superior to the rest of the models, without considering the long inference time, and the accuracy value is similar to less complex models.

## 5. Conclusions and Future Work

This study aims to analyze relevant aspects in developing a monitoring system based on constrained devices to classify acoustic patterns of bee colonies using CNN models. The performance, inference time, and energy efficiency of ten CNN models were analyzed in three SBCs: an Nvidia Jetson Nano, an Orange Pi 5, and a Raspberry Pi 5. Most of the CNN models analyzed were specifically designed for constrained devices. Furthermore, by segmenting a dataset with acoustic samples of 30 s, three datasets were generated with spectrograms of different durations, 10, 5, and 1 s, to assess the impact on the inference performance of the CNN models.

Using the dataset with 30 s samples, the CNN models achieved 96% accuracy. In models trained using datasets containing samples of 10 or 5 s, the accuracy decreased by around 1–2% compared to previous models trained with datasets containing larger acoustic samples. In the models trained with 1 s spectrograms, the reduction in accuracy is around 5%, and in all the models, the accuracy drops below 90%. In most cases, the dataset containing 5 s spectrograms provides enough learning to CNN architectures to keep the accuracy above 90% while saving memory space and reducing the preprocessing step.

Regarding the inference time results, all models showed similar values except for the ConvNeXt model, which had the highest inference times. Despite the complexity of the model, ConvNeXt achieved comparable accuracy to less complex architectures like EfficientNet or RegNet. In addition, the model SuffleNet achieved the fastest inference time with an accuracy value superior to other models. Therefore, less complex CNN models accomplish the performance of more complex models with only a negligible reduction in performance.

In most cases, the GPU included in the NJN provides superior performance, reducing the inference time and energy consumption. This feature makes the NJN an excellent option to implement a monitoring system. The downside of the NJN is the software is no longer supported, which is not a major problem for the RPi5. On the other hand, although the OPi5 does not include a GPU to accelerate CNN architectures, it achieves better inference times than the NJN, with models such as ShuffleNet, SqueezeNet, or AlexNet. Also, the OPi5 has approximately the same energy consumption as NJN in MAX mode, for example, in models such as ShuffleNet, AlexNet, Squeezenet, or RegNet. Despite having an octa-core processor, the OPi5 exhibits lower power consumption than the RPi5, which has the highest power consumption, featuring a quad-core processor. The RPi5, having the slowest inference time and the highest energy consumption, achieves a result similar to the SuffleNet model, with the same level of energy consumption as NJN and an inference time close to the OPi5.

Models such as EfficientNet, MobileNet, and ResNet18 have an excellent balance between performance, energy consumption, and inference time. ShuffleNet is the model with less energy consumption, faster inference time, and a performance classification superior to other models such as SqueezeNet, MNASNet, or AlexNet.

In future work, new samples will be recorded at a length of 5 s. The acoustic sample quality will be enhanced by increasing the frequency sampling and bit depth. The original samples were recorded at a 12-bit resolution with a sampling frequency of 4 KHz. Also, the original dataset is very limited in diversity, given that the acoustics were captured in 15 days on dry, sunny days, which is characteristic of the region. Therefore, the CNN models will be prone to overfitting with limited generalization capacity. Analyzing the beehive acoustic in different biological and climatic contexts will be necessary for testing a monitoring system under real-life conditions. Image transformation techniques for data argumentation will be studied to enhance the generalization capacity of the CNN models. We will propose a specific image transformation for datasets containing spectrograms of bee acoustic signals.

## Figures and Tables

**Table 1:** Hardware specifications of SBCs.

	Nvidia Jetson Nano (NJN)	Orange Pi 5B (OPi5)	Raspberry Pi 5 (RPi5)
Processor	ARM Cortex-A57 quad-core (1.43 GHz)	RK3588S octa-core (2.4 GHz) 4xCortex-A76 + 4xCortex-A55	Broadcom BCM2712 quad-core ARM Cortex-A76 (2.4 GHz)
GPU	Nvidia Maxwell 128 CUDA cores	ARM Mali-G610	VideoCore VII GPU
RAM	4 GB LPDDR4	4 GB LPDDR4X	4 GB LPDDR4X
Storage	Micro-SD 64 GB	32 GB eMMC	Micro-SD 64GB
Recommended power supply	5 V–2 A micro-USB 5 V–4 A barrel jack	5 V–4 A USB-C	5 V–5 A USB-C

**Table 2:** TC66C USB-C multimeter features.

	Voltage	Current
Range	0–30 V	0–5 A
Resolution	0.1 mV	0.01 mA
Accuracy	±0.05%	±0.1%

**Table 3:** CNN architectures.

Models	Version	Parameters (Millions)	GFLOPS
MNASNet	05	2.21	0.1
MobileNet	V2 20	3.5	0.3
SqueezeNet	1.1	1.24	0.35
EfficientNet	b0	5.28	0.39
RegNet	y400mf	4.34	0.4
AlexNet	-	61.1	0.71
ShuffleNet	V2 0.5x	1.36	0.82
GoogLeNet		6.62	1.5
ResNet	18 18	11.68	1.81
ConvNeXt	tiny	28.58	4.46

**Table 4:** Specification of operating systems, packages, and library versions used in the SBC.

	RPi5	OPi5	NJN
Operating System	Raspberry Pi OS (Bookworm v12)	Orange Pi OS (Bullseye v11)	Ubuntu (v20.04)
Python	3.11.2	3.9.2	3.8.10
PyTorch	1.13	2.0.1	1.13
Torchvision	0.14.1	0.15.2	0.14.0

**Table 5:** Search space for hyperparameter optimization.

Learning Rate	Batch Size	Optimizer
0.0001–0.01	16, 32, 64	SGD, Adam

**Table 6:** Results of the hyperparameter search.

Dataset		MobileNet	Resnet18	ConvNeXt	AlexNet	EfficientNet	MNASNet	SqueezeNet	RegNet	GoogLeNet	ShuffleNet
	lr	0.0002	0.003	0.0001	0.0012	0.00025	0.0015	0.0001	0.0002	0.00018	0.0013
D30	op	Adam	SGD	Adam	SGD	Adam	Adam	Adam	Adam	Adam	Adam
	bs	16	16	64	64	16	64	16	64	64	16
	lr	0.0003	0.00015	0.0001	0.0012	0.00021	0.0012	0.0001	0.0002	0.00017	0.0006
D10	op	Adam	Adam	Adam	SGD	Adam	Adam	Adam	Adam	Adam	Adam
	bs	16	16	64	64	32	16	32	64	32	32
	lr	0.0001	0.0024	0.00012	0.0014	0.0002	0.001	0.0001	0.0002	0.0001	0.0016
D5	op	Adam	SGD	Adam	SGD	Adam	Adam	Adam	Adam	Adam	Adam
	bs	32	16	32	64	32	16	64	32	32	16
	lr	0.0002	0.00013	0.0001	0.0011	0.00035	0.0028	0.0001	0.0002	0.00018	0.00058
D1	op	Adam	Adam	Adam	SGD	Adam	Adam	Adam	Adam	Adam	Adam
	bs	64	64	64	64	16	32	16	16	64	16

lr—learning rate, op—optimizer, bs—batch size.

**Table 7:** Maximum accuracy achieved in the test step.

Dataset	MNASNet	MobileNet	SqueezeNet	EfficientNet	RegNet	AlexNet	SuffleNet	GoogLeNet	ResNet18	ConvNeXt
D30	0.917	0.965	0.9346	0.972	0.9694	0.9174	0.9582	0.9658	0.9602	0.9756
D10	0.9176	0.9504	0.9104	0.964	0.9534	0.8986	0.9416	0.9526	0.9494	0.9588
D5	0.897	0.9238	0.8948	0.943	0.936	0.8848	0.923	0.9364	0.9276	0.946
D1	0.7596	0.863	0.8458	0.8828	0.8692	0.833	0.864	0.8688	0.8702	0.8922

## Data Availability

We made use of the publicly available dataset at https://data.mendeley.com/datasets/t9prmbmdfn, accessed on 6 September 2024.

## Abbreviations

The following abbreviations are used in this manuscript:

CNN	convolutional neural networks
SBC	single-board computer
NJN	Nvidia Jetson Nano
RPi5	Raspberry Pi 5
Opi5	Orange Pi 5
PC	personal computer
FLOPS	floating point operations per second
MFCC	Mel frequency cepstral coefficients
SVM	support vector machine
KNN	k-nearest neighbors
RF	random forest
OSBH	open-source beehives
GPU	graphic processing unit
STFT	short-time Fourier transform
SGD	stochastic gradient descent
CUDA	compute unified device architecture
